# Robot Localisation Using UHF-RFID Tags: A Kalman Smoother Approach †

**DOI:** 10.3390/s21030717

**Published:** 2021-01-21

**Authors:** Farhad Shamsfakhr, Andrea Motroni, Luigi Palopoli, Alice Buffi, Paolo Nepa, Daniele Fontanelli

**Affiliations:** 1 Department of Industrial Engineering, University of Trento, 38123 Trento, Italy; farhad.shamsfakhr@unitn.it; 2 Department of Information Engineering, University of Pisa, 56122 Pisa, Italy; andrea.motroni@ing.unipi.it (A.M.); paolo.nepa@unipi.it (P.N.); 3 Department of Engineering and Computer Science, University of Trento, 38123 Trento, Italy; luigi.palopoli@unitn.it; 4 Department of Energy, Systems, Territory and Constructions Engineering, University of Pisa, 56122 Pisa, Italy; alice.buffi@unipi.it

**Keywords:** Radio Frequency IDentification, Kalman smoother, robot localisation

## Abstract

Autonomous vehicles enable the development of smart warehouses and smart factories with an increased visibility, flexibility and efficiency. Thus, effective and affordable localisation methods for indoor vehicles are attracting interest to implement real-time applications. This paper presents an Extended Kalman Smoother design to both localise a mobile agent and reconstruct its entire trajectory through a sensor-fusion employing the UHF-RFID passive technology. Extensive simulations are carried out by considering the smoother optimal-window length and the effect of missing measurements from reference tags. Monte Carlo simulations are conducted for different vehicle trajectories and for different linear and angular velocities to evaluate the method accuracy. Then, an experimental analysis with a unicycle wheeled robot is performed in real indoor scenario, showing a position and orientation root mean square errors of 15 cm, and 0.2 rad, respectively.

## 1. Introduction

The growth of Industry 4.0 applications lays the groundwork for the development of new services and solutions [1]. In this scenario, the lion’s share is likely to be taken by a new generation of autonomous vehicles, capable of operating in complex and dynamic industrial scenario with high flexibility and reliability. One of the most critical functionalities needed to enable these new services is the so-called localisation, defined as the ability to maintain an accurate estimate of each robot pose in time [2].

Since the large majority of production plants are actually indoor, localisation is almost entirely intended as indoor localisation [3,4]. Given the absence of a reliable Global Position System inside a plant or an indoor logistic area, several technologies have been proposed to deliver a reliable localisation service. Laser Imaging Detection and Ranging (LiDAR) [5], cameras [6,7], ultrasounds [8] or Radio Frequency (RF) systems [9] are available options used in the past. In the last category, particularly fall solutions with different performance and costs such as Wi-Fi [10], Bluetooth [11], Ultra Wide Band (UWB) [12,13] or RF Identification (RFID) [14,15] technologies. The ultra-high-frequency (UHF) RFID technology is gaining traction due to its being multipurpose (a RFID tag can be used both to identify a pallet and to localise it), to its low-cost, and to its easy installation and maintenance. The system operates with radio-waves in the band between 860 MHz to 960 MHz, which can penetrate through many materials without significant alterations. This feature provides the system with an intrinsic level of robustness, and marks a remarkable difference with respect to technologies operating at higher frequencies or adopting line-of-sight equipments, such as LiDARs. As an additional remark, the use of RFID makes the system immune from any type of privacy concerns, which could be present in camera-based systems.

Two types of solutions have been conceived in RFID-based vehicle localisation. The first category exploits an infrastructure of fixed reader antennas, with the robot being equipped with an RFID tag [16]. The second one, on the contrary, is based on having the vehicle equipped with a reader connected to one or more antennas, while a grid of RFID tags is deployed in the surrounding scenario at known locations [17]. In case of unknown reference tag positions, simultaneous localisation and mapping (SLAM) systems has been also designed [18]. For sure, the second class of solutions is arguably more appealing to a possible implementer since the passive UHF-RFID tags do not require direct power supply (contrary to the UWB nodes).

The data association can be easily performed thanks to the tag unique identifier (Electronic Product Code, EPC). Moreover, UHF-RFID tags can be easily installed at the floor side [19], at the ceiling [18] or in almost any other place in the considered environment [20]. In harsh industrial scenarios with lots of metallic structures, Commercial-Off-The-Shelf (COTS) on metal tags can be employed. Finally, when the vehicle is natively equipped with COTS RFID hardware, e.g., the RFID-robot employed for inventory tasks [21], the implementation of the RFID-based localisation infrastructure amounts just to reference tag deployment.

A detailed survey of solutions based on the RFID technology for vehicle localisation has been presented by Motroni et al. in [22]. At the state-of-the-art, systems rely only on the employment of an RFID infrastructure or on sensor-fusion solutions employing both RFID technology with proprioceptive sensors [17] or both RFID systems with other exteroceptive sensors [23]. Measured data are typically combined through dynamic estimation algorithms based for example on Kalman filters or Particle filters. The most representative and investigated category is based on UHF-RFID systems combined with proprioceptive sensors, e.g., encoders, Inertial Measurement Units (IMUs) or optical flow sensors. Generally speaking, a sensor-fusion approach increases the localisation accuracy and reduces the reference tags density in the infrastructure [20].

In this paper, we propose the employment of a Rauch-Tung-Striebel (RTS) smoother based on an Extended Kalman Filter (EKF) to determine the best possible estimate of the whole trajectory followed by an agent inside a plant. This issue stems from an actual industrial application where the estimate of the set of trajectories followed by the available moving agents is of major relevance (either if they are autonomous or actually controlled by human workers). This knowledge is extremely important to the plant manager to detect points of failure or of possible collisions as well as to optimise the team trajectories as a whole. The method first proposed in [24] is here retrieved and deeply characterised through both a numerical analysis and an experimental validation with a real mobile robot in an indoor scenario. The solution robustness is discussed when measurements are missing, important feature which is hardly mentioned in the literature, showing accurate localisation of the proposed smoother with respect to filter-based approaches. After a description of the related work in Section 2, the smoother design is described in Section 3 together with the experimental setup. Then in Section 4, we discuss and report on a new set of experimental results that proves the applicability and the effectiveness of the proposed approach on an current set-up.

## 2. Related Work

To estimate the agent position assuming the knowledge of the passive tag locations in a reference frame, both amplitude and phase of the signal backscattered by the tags and received at the reader side can be profitably used [19,25]. Several techniques based on the Received Signal Strength Indicator (RSSI) have been proposed, being the more widespread parameter available in commercial readers [19,26,27].

In [27], a UHF-RFID system was presented to navigate a robot through a guided path. The robot pose was estimated through a Particle Swarm Optimisation (PSO) algorithm applied to the RSSI data, and the navigation was conducted through a Fuzzy Logic Controller (FLC). A numerical analysis showed that the proposed method is affected by a RMSE of 6 cm when the robot moves in a 36 m${}^{2}$ room with two reference tags. In [19], the localisation of a mobile robot equipped with two reading antennas is proposed. The method is based on the principle that if an RFID tag is located at the same distance between the two antennas, the correspondent RSSI measurements should ideally be identical. The method shows a centimetre order localisation error for a robot travelling at different speeds in a real indoor scenario of 5×10 m${}^{2}$ and exploiting 578 reference tags.

However, the signal phase is by far more robust and effective for localisation purposes especially in indoor scenarios [21]. The main challenge to be solved is the ambiguity of phase measurements due to the 2π-periodicity [28], and the knowledge of the phase offset term. The latter is typically solved through the employment of the Phase Difference of Arrival (PDoA) [29]. Indeed, unwrapping techniques [30] or phasor use [31] can face and solve the phase ambiguity issue.

In [32], the robot localisation was solved through a Multi-Hypothesis Extended Kalman Filter that combines data acquired from two rotary encoders and RFID phase data gathered by a set of reference tags placed in known locations at the ceiling. Tags were designed with an ad hoc radiation pattern. The method requires for a pre-calibration procedure to estimate the offset term typically affecting the phase measurements. Such calibration procedure is then avoided thanks to the method extension proposed by the same authors in [33], where the RSSI data are employed as additional input parameters. Moreover, such a modification allows reducing the accuracy required for the knowledge of the reference tag positions. According to the experimental results, an average localisation error of centimetre order was obtained within a 4×3 m${}^{2}$ room by employing two reference tags.

In [20], a novel phase-based sensor-fusion tracking method for moving agents was presented after the European patent application [34]. The robot localisation is achieved through a Synthetic Aperture Radar (SAR) approach [29] by collecting consecutive phase measurements along the agent trajectory with respect to the reference tags. Such an approach allows reducing the density of the reference-tag infrastructure, which can be installed at any position within the indoor scenario. Then, the phase data are combined with the kinematic data collected by odometers, through a sensor-fusion technique. The obtained average localisation error is around 11 cm for a 4 m long trajectory in an indoor office environment by employing two reference tags. The method capability has been validated when odometry measurements are available, but it can be adapted to work with any kind of proprioceptive sensors. Furthermore, it works with COTS devices, and does not require for a calibration procedure.

In [35], a particle filter was presented to track a mobile robot equipped with two rotary encoders and two RFID antennas facing to the floor. The algorithm exploits the PDoA gathered by an infrastructure of reference tags deployed on the floor. Experiments were conducted in a 5×5 m${}^{2}$ office environment with the presence of metallic objects, with 42 tags deployed on the floor and arranged as a grid with spacing of 60 cm. The obtained median position error was about 6 cm.

It is worth mentioning the work presented in [18], where an indoor simultaneous localisation and mapping (SLAM) problem is considered. A set of passive tags was deployed at the ceiling in unknown locations. RFID phase measurements are fused with the robot odometry to determine both the robot pose and the tag coordinates through a Rao-Blackwellized Particle Filtering approach. Experimental results show a global mapping error of a few centimetres in a 3.5×2 m${}^{2}$ laboratory environment with six tags.

In [36], Magnago et al. showed that the localisation problem can be theoretically solved locally, i.e., the a priori starting position estimate is closer to the initial actual position of the agent, and considering unicycle-like robots, which are a commonplace in industrial environments for warehouse management or for transportation in production lines. Successively, it has been shown that the problem can be actually solved also globally [37], provided that some mild assumptions on the uncertainties are satisfied. Given that the localisation problem is well-posed when RFID tags are used and fused with odometry for unicycle-like vehicles [37], the authors of this paper recently proposed in [24] a solution for industrial IoT applications based on an Extended Kalman Filter. The method has been conceived for industrial scenarios and account for specific position constraints. As an example, the issue related to pallet handling in warehouse applications [38] requires a pallet-placing uncertainty in the order of a few centimetres with a high level of confidence [39]. Hence, the main objective of the proposed approach is to robustly reduce to the maximum extent the localisation uncertainty and the vehicle trajectory estimation uncertainty to meet the industrial requirements.

## 3. Materials and Methods

A configuration of a unicycle robot with a state vector $s={\left[x,\;y,\;\theta \right]}^{T}$ has the following form:

(1) $$\begin{array}{cc} \dot{x} & =v\cos\theta \\ \dot{y} & =v\sin\theta \\ \dot{\theta } & =\omega , \end{array}$$

where θ is the orientation with respect to the $X_{w}$ axis of the reference frame 〈W〉, while *v* (the forward velocity) and ω (the angular velocity) are the input variables. Assuming that the RFID readings are measured with period $T_{s}$ and that the command variable *v* and ω are approximately constant throughout the sampling period, it is possible to find the following discrete time Zero-order-Hold equivalent dynamics [40]

(2) $$\begin{array}{cc} x_{k+1} & =\left\{\begin{array}{cc} x_{k}+v_{k}T_{s}\cos\theta_{k} & \mathrm{if}\;\omega_{k}=0,\\ x_{k}+2\frac{v_{k}}{\omega_{k}}\sin\left(\frac{\omega_{k}}{2}T_{s}\right)\cos\left(\theta_{k}+\frac{\omega_{k}}{2}T_{s}\right) & \mathrm{otherwise}, \end{array}\right.\\ y_{k+1} & =\left\{\begin{array}{cc} y_{k}+v_{k}T_{s}\sin\theta_{k} & \mathrm{if}\;\omega_{k}=0,\\ y_{k}+2\frac{v_{k}}{\omega_{k}}\sin\left(\frac{\omega_{k}}{2}T_{s}\right)\sin\left(\theta_{k}+\frac{\omega_{k}}{2}T_{s}\right) & \mathrm{otherwise}, \end{array}\right.\\ \theta_{k+1} & =\theta_{k}+\omega_{k}T_{s}. \end{array}$$

where we made use of the simplified notation $x_{k+1}=x\left(\left(k+1\right)T_{s}\right)$ (i.e., $s_{k+1}=[x_{k+1},y_{k+1},$ $\theta_{k+1}{]}^{T}$).

### 3.1. Signal Model

RFID-based localisation systems comprise a set of RFID tags deployed at known locations inside a certain environment and an RFID reader that is able to read the backscattered signal. The phase delay of the RFID signal with 2π-radian period can be stated as

(3) $$\begin{array}{c} \phi_{i}=\frac{4\pi d_{i}}{\lambda }+\delta_{i}^{\phi }=\phi_{i}^{\prime }+2\pi N+\delta_{i}^{\phi }, \end{array}$$

where λ is the wavelength, $d_{i}$ is the actual distance between the tag and the reader, and $\delta_{i}^{\phi }$ is an offset due mainly to cables, reader and antenna components and transponder backscattering [28]. Moreover, $\phi_{i}^{\prime }=\mathrm{mod}\left(\phi_{i},2\pi \right)$ where *N* is the integer number of half-wavelengths within the distance $d_{i}$. In practice, the unwrapped absolute phase $\phi_{i}$ cannot be measured directly. Therefore, *N* is unknowable and the phased-based measured distance $d_{i}^{m}$ is inherently ambiguous, i.e.,

(4) $$d_{i}^{m}=d_{i}+\delta_{i}^{d}=\frac{\phi_{i}^{\prime }}{4\pi }\lambda +\delta_{i}^{d},$$

where the uncertainty term $\delta_{i}^{d}=N\frac{\lambda }{2}+\frac{\lambda }{4\pi }\delta_{i}^{\phi }$ in general can be very large, but it is also approximately constant for distance variations smaller than half wavelength. Therefore, since ${\dot{\delta }}_{i}^{d}\approx 0$ the variation in time of the relative distance between the RFID reader and the *i*-th tag tends to be insensitive to phase ambiguity and offset, and it can be written as

(5) $${\dot{d}}_{i}\;=\;{\dot{d}}_{i}^{m}\;\approx \;\frac{\lambda }{4\pi }{\dot{\phi }}_{i},$$

We denote the actual distance of the *i*-th tag at time $kT_{s}$, where $T_{s}$ is the sampling period of the RFID reader, as $d_{i,k}$, i.e.,

(6) $$\begin{array}{c} d_{i,k}=\sqrt{{\left(X_{i}-x_{k}\right)}^{2}+{\left(Y_{i}-y_{k}\right)}^{2}}, \end{array}$$

where $\left(X_{i},Y_{i}\right)$ are the Cartesian coordinates of the RFID tag. Thus, the time derivative of the distance function (6) and using (4) is

(7) $$\begin{array}{c} {\dot{d}}_{i,k}={\dot{d}}_{i,k}^{m}=v_{k}\left[\frac{\left(x_{k}-X_{i}\right)\cos\left(\theta_{k}\right)+\left(y_{k}-Y_{i}\right)\sin\left(\theta_{k}\right)}{d_{i}}\right], \end{array}$$

which is the instantaneous variation of the distance at time $kT_{s}$. Albeit (7) is inherently robust to the phase ambiguity, its validity is useful for localisation only locally, i.e., for displacements taking place in a neighbourhood of a known initial location [36]. The adoption of the EKF-RTS smoother proposed in this paper alleviates this issue by incorporating two consecutive robot positions, i.e., $s_{k}$ and $s_{k-1}$, in the filter state, as better shown later. As a consequence of this choice, the output function adopted is a straightforward discrete approximation of (7), i.e., a finite difference of the measured distances (4). More precisely, by denoting with $d_{i,k}^{m}$ and $d_{i,k}$ the measured and actual distances at time $kT_{s}$ from the *i*-th tag, respectively, we have that the adopted output function for the *i*-th tag is

(8) $$\begin{array}{c} h_{i}\left(s_{k},s_{k-1}\right)=d_{i,k}^{m}-d_{i,k-1}^{m}=d_{i,k}-d_{i,k-1}. \end{array}$$

### 3.2. Filter Design

We are interested in determining an estimate of the state of an unicycle-like vehicle (very common in, e.g., industrial autonomous warehouses) ${\hat{s}}_{k}$ at time $kT_{s}$ fusing the sensor readings coming from the wheel encoders and the RFID tag ranging. The odometry measurements are related to the linear and angular velocities increment of the vehicle $v_{k}T_{s}$ and $\omega_{k}T_{s}$, respectively, and detailed in (2). The UHF-RFID tag sensed quantities are described in (4) and denoted with $d_{i,k}^{m}$, which are assumed to be collected up to time $kT_{s}$.

Moreover, we are interested in having an effective estimate of the overall trajectory ${\hat{s}}_{0,\ldots ,k}$ from time 0 to $kT_{s}$ using all the measurements up to time $kT_{s}$, i.e., *full-length* smoother named SEKF${}^{2}$, or the measurements in the interval $\left[\left(k-n\right)T_{s},kT_{s}\right]$, where n>0 is the length of a fixed-lag in the *fixed-lag* smoother (SEKF) and represents a tuning parameter that should be optimally determined.

In particular, we will focus on an EKF-RTS Smoother estimator for robot positioning through globally observable bended trajectories, i.e., trajectories having $\omega_{k}\neq 0$ for a sufficiently long time as derived in [37]. The robot moves in an indoor scenario instrumented with several M≥3 RFID tags in known positions. At each time step $kT_{s}$, the vehicle receives a measurement (3), with an unknown offset. The offset-free RFID phase measurement (8) described in Section 3 are organised in a vector of finite difference of measurements, i.e.,

(9) $$h\left(s_{k},s_{k-1}\right)=\left[\begin{array}{c} h_{1}\left(s_{k},s_{k-1}\right)\\ h_{2}\left(s_{k},s_{k-1}\right)\\ \ldots \\ h_{M}\left(s_{k},s_{k-1}\right) \end{array}\right]=\left[\begin{array}{c} d_{1,\;k}-d_{1,\;k-1}\\ d_{2,\;k}-d_{2,\;k-1}\\ \ldots \\ d_{M,\;k}-d_{M,\;k-1} \end{array}\right].$$

Due to (9), the state space of the filter is represented by

$${\hat{q}}_{k}={\left[{\hat{s}}_{k},{\hat{s}}_{k-1}\right]}^{T}={\left[{\hat{x}}_{k},\;{\hat{y}}_{k},\;{\hat{\theta }}_{k},\;{\hat{x}}_{k-1},\;{\hat{y}}_{k-1},\;{\hat{\theta }}_{k-1}\right]}^{T},$$

whose dynamic is given by

(10) $${\hat{q}}_{k+1}=f\left({\hat{q}}_{k},u_{k},\gamma_{k}\right)=\left[\begin{array}{c} f_{1}\left({\hat{s}}_{k},u_{k},\gamma_{k}\right)\\ f_{2}\left({\hat{s}}_{k},u_{k},\gamma_{k}\right)\\ f_{3}\left({\hat{s}}_{k},u_{k},\gamma_{k}\right)\\ {\hat{x}}_{k}\\ {\hat{y}}_{k}\\ {\hat{\theta }}_{k} \end{array}\right]$$

where $u_{k}={\left[v_{k},\omega_{k}\right]}^{T}$ is the vector of the model velocities, while $\gamma_{k}={\left[\gamma_{v_{k}},\gamma_{\omega_{k}}\right]}^{T}$ are the noises affecting the model velocities estimate given the odometry and assumed zero-mean, white and normally distributed with diagonal covariance matrix $Q_{k}$. Finally, $f_{i}\left({\hat{s}}_{k},u_{k},\gamma_{k}\right)$ are the system model dynamic expressed in (2). In a standard EKF, the predicted model uncertainty will be given by the respective Jacobians, i.e.,

(11) $$\begin{array}{cc} P_{k+1}^{-} & =A_{k}P_{k}A_{k}^{T}+B_{k}Q_{k}B_{k}^{T}, \end{array}$$

with

$$A_{k}=\left[\begin{array}{cccccc} 1 & 0 & \frac{\partial f_{1}}{\partial \theta_{k}} & 0 & 0 & 0\\ 0 & 1 & \frac{\partial f_{2}}{\partial \theta_{k}} & 0 & 0 & 0\\ 0 & 0 & 1 & 0 & 0 & 0\\ 1 & 0 & 0 & 0 & 0 & 0\\ 0 & 1 & 0 & 0 & 0 & 0\\ 0 & 0 & 1 & 0 & 0 & 0 \end{array}\right]\mathrm{and}\;B_{k}=\left[\begin{array}{ccc} \frac{\partial f_{1}}{\partial v_{k}} & \frac{\partial f_{1}}{\partial \omega_{k}}\\ \frac{\partial f_{2}}{\partial v_{k}} & \frac{\partial f_{2}}{\partial \omega_{k}} & \\ 0 & T_{s}\\ 0 & 0\\ 0 & 0\\ 0 & 0 \end{array}\right],$$

where the superscript $\cdot^{-}$ notation is used to denote the predicted estimates given the model. Of course, the initial value for the state is $q_{0}$, while the initial covariance is $P_{0}$.

Since the measurement function is modelled as (9), we have in the filter

(12) $$z_{k}=h\left({\hat{s}}_{k},{\hat{s}}_{k-1}\right)+\varepsilon_{k}=h\left({\hat{q}}_{k}\right)+\varepsilon_{k},$$

where $\varepsilon_{k}$ is the measurement uncertainty, modelled as a zero-mean, white and normally distributed sequence with covariance $R_{k}$. Hence, the EKF equations for the estimates updates

(13) $$\begin{array}{cc} K_{k} & =P_{k}^{-}H_{k}^{T}{\left(H_{k}P_{k}^{-}H_{k}^{T}+R_{k}\right)}^{-1},\\ {\hat{q}}_{k} & ={\hat{q}}_{k}^{-}+K_{k}\left(z_{k}-h\left({\hat{q}}_{k}^{-}\right)\right),\\ P_{k} & =\left(I-K_{k}H_{k}\right)P_{k}^{-}, \end{array}$$

where $K_{k}$ is the Kalman gain, $H_{k}\in R^{M\times 6}$ is the Jacobian of the measurement function (9), i.e.,

(14) $$H_{k}=\frac{dh({\hat{q}}_{k})}{d{\hat{q}}_{k}}=\left[\begin{array}{cc} \frac{\partial d_{1,\;k}}{\partial s_{k}} & -\frac{\partial d_{1,\;k-1}}{\partial s_{k-1}}\\ \frac{\partial d_{2,\;k}}{\partial s_{k}} & -\frac{\partial d_{2,\;k-1}}{\partial s_{k-1}}\\ \vdots & \vdots \\ \frac{\partial d_{M,\;k}}{\partial s_{k}} & -\frac{\partial d_{M,\;k-1}}{\partial s_{k-1}} \end{array}\right].$$

#### EKF Smoother

The EKF is a filter, hence it is able to retrieve the state ${\hat{s}}_{k}$ using the measurements up to time $kT_{s}$, i.e., the first part of the problem defined in Section 3.2. For the second, we make use of the RTS smoother [41], which builds up a backward recursion for vehicle trajectory estimate, as detailed next:

(15) $$\begin{array}{cc} {\hat{q}}_{k+1}^{-} & =A_{k}q_{k},\\ P_{k+1}^{-} & =A_{k}P_{k}A_{k}^{T}+B_{k}Q_{k}B_{k}^{T},\\ G_{k} & =P_{k}A_{k}^{T}{\left[P_{k+1}^{-}\right]}^{-1},\\ {\hat{q}}_{k}^{s} & ={\hat{q}}_{k}+G_{k}\left[{\hat{q}}_{k+1}^{s}-{\hat{q}}_{k+1}^{-}\right],\\ P_{k}^{s} & =P_{k}+G_{k}\left[P_{k+1}^{s}-P_{k+1}^{-}\right]G_{k}^{T}, \end{array}$$

where $G_{k}$ is the smoother gain and where we used the superscript *s* to denote the smoothed quantities.

**Figure 1:**
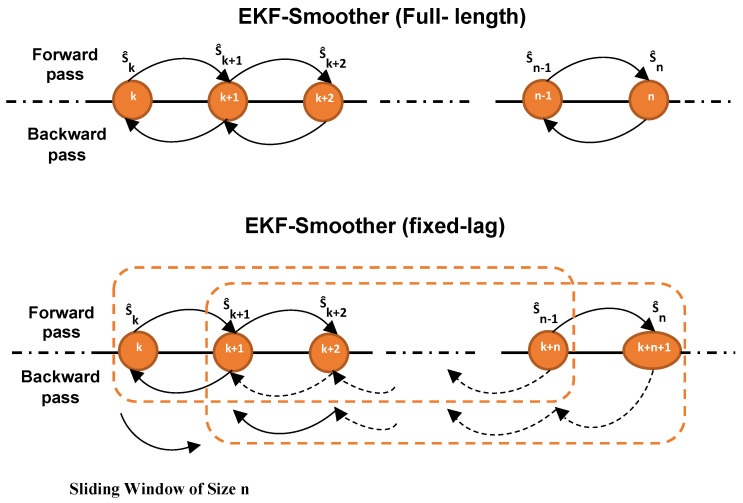
The process of the (**Top**) EKF-Smoother fixed-lag and (**Bottom**) EKF-Smoother Full-length algorithms.

Furthermore, we have implemented two different types of smoother. As depicted in Figure 1, the forward filter is responsible for processing the RFID phase samples sequentially, and the backward filter augment the sample estimation reversely to compensate for the estimation error of the EKF. In the fixed-lag smoother (see Figure 1, bottom), for estimating the smoothed value at time step *k*, only the measurements between k+1 and k+n+1 are used, where *n* is a fixed-lag (i.e., a fixed number of phase samples to be smoothed). Therefore, for each smoothed estimate ${\hat{q}}_{k}^{s}$, an adaptive window of *n* RFID phase measurements is considered. Thus, the smoothed values for all time steps can be achieved using a sliding window of size *n* in near real-time [42]. Another approach instead considers all the values, thus letting *n* be the sequence of *all* the available measurements (see Figure 1, top). Obviously this strategy is much more computationally demanding compared to fixed-lag smoother, and might not be affordable in real time.

### 3.3. Experimental Setup

**Figure 2:**
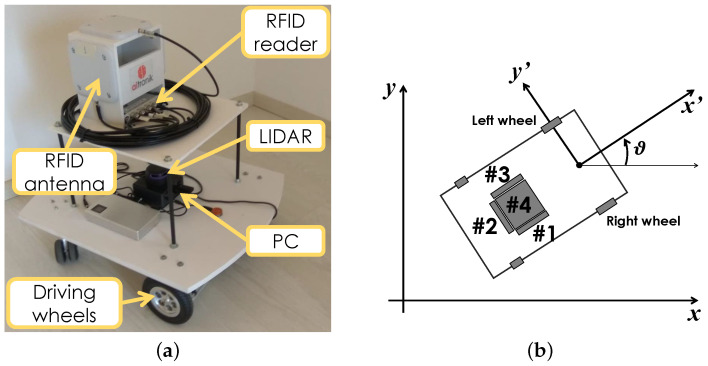
(**a**) Robot used for the experimental campaign with its different components. (**b**) Schematic representation of the robot (top view). The robot antennas are denoted with the symbols #1, #2, #3, and #4.

For the experimental campaign, we used a self-made unicycle wheeled mobile robot with a differential front-drive kinematics as the one reported in (2), depicted in Figure 2a and built by the Italian Company AITRONIK srl.

The robot axle length is equal to $l_{a}=0.53$ m and the height is h=0.68 m. The robot is equipped with two commercial rotary encoders belonging to the Parallax Arlo Robot kit. The encoders operate with 144 pulses for a full tire revolution and 100 Hz acquisition frequency. Since the robot tires have a diameter of 152 mm, the induced quantisation step on the linear displacement is around 3.3 mm. The robot is also equipped with an Impinj Speedway R420 UHF-RFID reader configured to transmit a signal with a frequency $f_{0}=865.7$ MHz (ETSI channel #4). It is connected with four Time-7 Compact Outdoor RAIN RFID circularly polarised (CP) antennas with HPBW = $115^{\circ }$ and Gain=5 dBiC, where “dBiC” stands for the decibel above the gain of a CP isotropic antenna. Each antenna faces a different area as shown from the top view in Figure 2b. All of them are placed at a height of 0.58 m from the ground, except antenna #4 which is placed at a height 0.68 m. The robot adopted in the experiments can be driven manually through a remote control, i.e., a joystick. Since we are interested in actual industrial applications, where the estimate of the set of trajectories followed by the available moving robots is of major relevance either if they are autonomous or actually controlled by human workers, and just focused on the trajectory estimation (how the localisation uncertainty acts on the robot control law can be found, e.g., in [43,44]), we decided to steer the robot in the environment using the remote controller.

To validate the method performance, the ground truth data of the vehicle trajectory are computed through a state-of-the-art SLAM algorithm by acquiring data from a Slamtec RP LIDAR A34 Laser Range Finder (LRF). All the payload-sensors are power supplied with two batteries and properly synchronised in time, and a small on-board PC is employed to gather and transmit all the data to an external laptop PC via a Wi-Fi connection.

**Figure 3:**
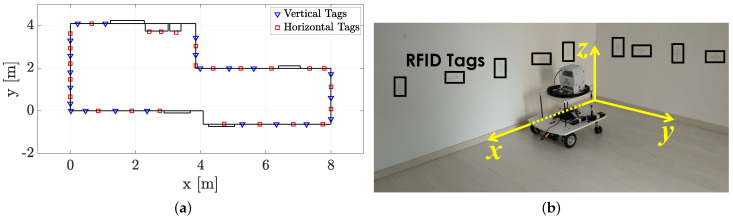
Experimental setup. (**a**) Room map, with the two areas. Vertical tags are depicted as blue triangular markers, Horizontal tags are depicted as red square markers. (**b**) A picture of the robot taken during the experimental campaign.

The test area consists of a single-room office-like environment with a total size of around 26 m, divided in two areas of 3.85×4.10 m${}^{2}$ and 3.90×2.60 m${}^{2}$, respectively, as shown in Figure 3a.

47 EasyRFID Bone reference tags with Monza R6 chip are placed according to a not regular spacing along the room walls (Figure 3b) at a height of 0.70 m from the floor. The average tag spacing on the *x*-direction is 0.53 m, whereas the tag spacing on the *y*-direction is 0.35 m (see Figure 3b). The tags were placed both with vertical and horizontal orientations with respect to the ground plane to reduce the mutual electromagnetic coupling among them (tags are represented with triangular and squared markers in Figure 3b). During the experiments only antenna #1 (indicating in Figure 2a) is on. With respect to the robot local reference frame, the antenna #1 points the negative direction of the *y*-axis (the right side, see Figure 2b), and its displacement with respect to the robot rotation centre is $\Delta_{\#1}={\left[-0.31,-0.11\right]}^{T}$ m.

## 4. Results

Firstly, the performance of the proposed EKF-RTS for localisation in indoor industrial environments is investigated and compared with the classical EKF through a series of simulations. In particular, extended simulations and tests in a variety of scenarios and conditions have been carried out to highlight the behaviour and the sensitivity of the proposed solution in different operating conditions. Then, extensive experiments on an actual deployment and with an actual vehicle have been shown to certify the applicability and the effectiveness of the solution.

### 4.1. Simulation Results

**Figure 4:**
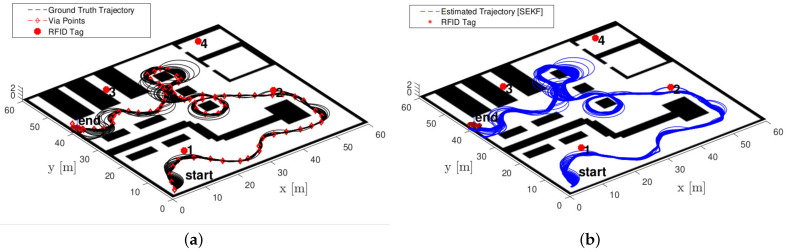
Trajectory samples generated by different velocities for the same path in a realistic warehouse scenario. (**a**) Ground truth trajectories, (**b**) estimated trajectories by the SEKF. A possible configuration of the four RFID is reported with red circle markers and denoted with numbers.

In order to validate the accuracy of the proposed strategy, in the first simulation scenario, we carried out Monte Carlo simulations with 10,000 trials, i.e., 100 trials over 10 different trajectories and under 10 random deployment of the *M* RFID tags and assuming no constraints on the tag placement. The different trajectories are synthesised automatically using sequences of randomly generated via-points. All of the simulations are conducted in a simulated warehouse environment using Robotics System Toolbox in Matlab (see Figure 4).

In all the trials, four RFID tags, with random locations (see an example in Figure 4) are considered. The system evolution along each trajectory is simulated with a sampling time $T_{s}=200$ ms. In each trajectory, the desired linear velocity $v_{k}$ and the maximum angular velocity $\omega_{k}$ of the mobile platform moving on the plane (and explicitly reported in (2)) are set equal to 2.8 m/s while the controlled angular velocity depends on the shape of the trajectory and ranges in the set (−0.5,0.5) rad/s. All the uncertainties (i.e., $\gamma_{k}$ acting on the vehicle velocities in (10), and $\varepsilon_{k}$ acting on the offset-affected RFID ranges in (12)) are assumed normally distributed, zero-mean and white. Therefore, $\gamma_{k}∼N\left(0,Q_{k}\right)$, where $Q_{k}=\mathrm{diag}\left(\sigma_{v}^{2},\sigma_{\omega }^{2}\right)$ and $\sigma_{v}=0.08$ m/s and $\sigma_{\omega }=0.09$ rad/s, which are a conservative assumption with respect to an actual industrial vehicle. Similarly, $\varepsilon_{k}∼N\left(0,R_{k}\right)$, where $R_{k}=2\sigma_{r}^{2}I_{M}$ and, hence, $\sigma_{r}=0.1$ m is the range reading uncertainty. We assume here that $I_{M}$ is the identity matrix of dimension *M* (i.e., of the same dimension of the number of available RFID tags). As reported in (2), the vehicle state is identified with its Cartesian coordinates $\left(x_{k},y_{k}\right)$ and its orientation $\theta_{k}$. Therefore, the covariance matrix expressing the vehicle initial location uncertainty is $P_{0}=\mathrm{diag}(0.1^{2}\;m^{2},0.1^{2}\;m^{2},0.1^{2}\;{\mathrm{rad}}^{2}$. By denoting with $s_{k}={\left[x_{k},y_{k},\theta_{k}\right]}^{T}$ the vehicle state at time $kT_{s}$, the initial state estimate is assumed to be ${\hat{s}}_{0}=s_{0}+\eta$, where $\eta ∼N(0,P_{0})$. Notice that we denote with the hat $\hat{\cdot }$ the quantities that are estimated.

#### 4.1.1. Optimal Window Length

**Figure 5:**
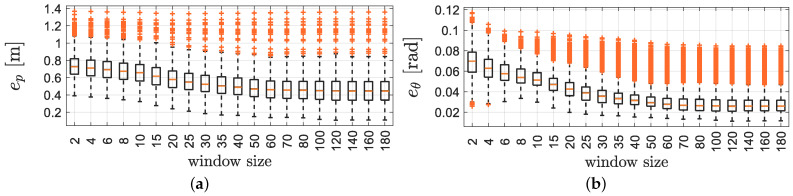
The RMSE for position (**a**) and orientation (**b**) of the SEKF with different window sizes.

Since the length of the fixed lag (i.e., the measurement window size) of the SEKF can be tuned and affects its performance, we first need to figure out if there is a way to optimally determine such a window size, which asks for the determination of a quantitative cost index. Since the performance are related to the estimation error that can be obtained, we choose the Root Mean Squared Error (RMSE) for position and orientation as a figure of merit, i.e.,

$$e_{p}^{i}=\sqrt{\frac{\sum_{k=1}^{N_{i}}{\left({\hat{x}}_{k}-x_{k}\right)}^{2}+{\left({\hat{y}}_{k}-y_{k}\right)}^{2}}{N_{i}}},$$

and

$$e_{\theta }^{i}=\sqrt{\frac{\sum_{k=1}^{N_{i}}{\left({\hat{\theta }}_{k}-\theta_{k}\right)}^{2}}{N_{i}}},$$

where $N_{i}$ is the number of samples for the *i*-th Monte Carlo execution. Therefore, we first analyse the effect of the window size by computing thebox-and-whiskers of Figure 5 for the different trajectories with different window lengths (from 2 to 180 samples) and assumingthe detection of four RFID tags.

**Figure 6:**
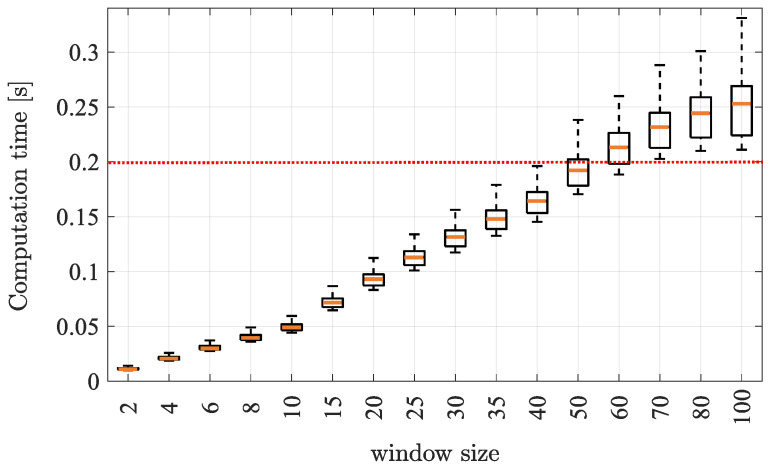
Computation time for different window sizes of the SEKF. The horizontal dashed line is the available sampling time.

Of course, while the window length is inversely proportional to the uncertainty, it is actually directly proportional to the computational burden. In particular, Figure 6 reports the computation time of the SEKF as a function of the window length.

These results are collected using a 1.70-GHz Intel Core i3 microprocessor endowed with 8 GB RAM. It is now evident from Figure 5 and Figure 6 that the higher is the computation time, the lower is the localisation error (up to 100 samples). To select the window size, we consider the sampling time $T_{s}=200$ ms selected at the beginning of this section as a threshold for a real-time execution of the algorithm (i.e., the computation should end before the next data arrives), hence the window length has been fixed to n=55 samples.

#### 4.1.2. Comparative Analysis

**Figure 7:**
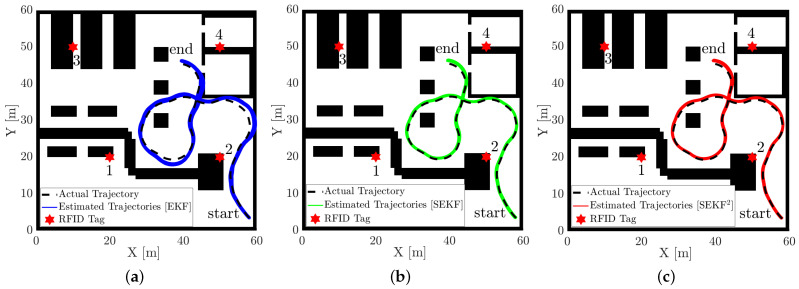
Sampled estimated trajectories by (**a**) EKF, (**b**) SEKF and (**c**) SEKF${}^{2}$ based on the four RFID tags reported in the figure with star markers.

We present now a comparative analysis between a standard EKF, the SEKF and the SEKF${}^{2}$ reported using four RFID tags only. As stated above, the fixed-lag SEKF window has been fixed to n=55 samples. The length of the trajectories (i.e., the number of available measurements) used are different and range from 180 to 270 samples, i.e., about 36 to 54 s. A qualitative analysis using one actual trajectory is proposed in Figure 7.

**Figure 8:**
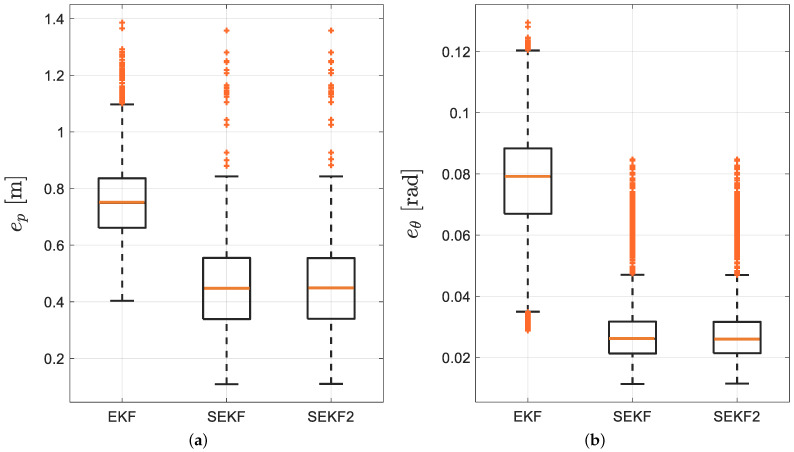
(**a**) Position and (**b**) orientation RMSE box-and-whiskers for EKF, SEKF and SEKF${}^{2}$, respectively.

It has to be noted how visually the SEKF and SEKF${}^{2}$ performs better than the EKF, as expected, while mild differences exist among them. This fact is further substantiated by the RMSE quantitive comparisons among the three different algorithms reported in terms of the box-and-whiskers plot in Figure 8.

It is now quantitatively evident how the smoother (either SEKF or SEKF${}^{2}$) ensures a lower error for the trajectory estimates. As reported in Figure 6, being the SEKF${}^{2}$ unfeasible in real time but yielding de facto the same uncertainties of SEKF, the rest of the results report the latter only.

#### 4.1.3. Filter Robustness

**Figure 9:**
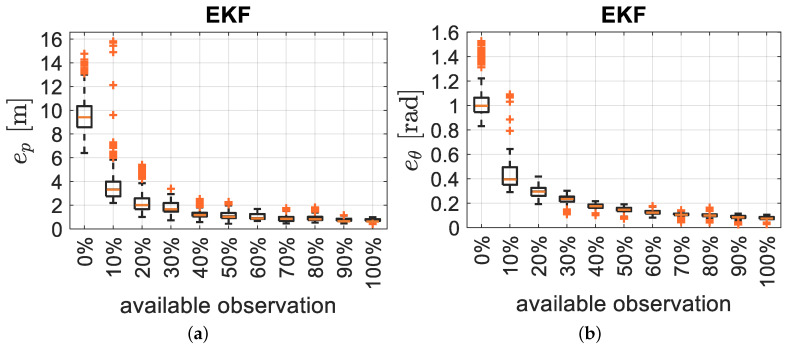

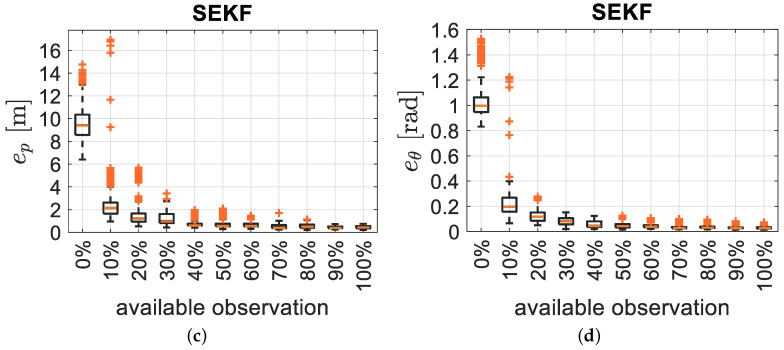
(**a**,**c**) Position and (**b**,**d**) orientation RMSE for different percentages of available observations for the EKF (**a**,**b**) and the SEKF (**c**,**d**).

We investigate here the impact of the availability of the measurements for the pose estimation by considering a variable percentage of the received information at each time step through Monte Carlo analysis with 1200 trials. Using four RFID tags, the minimum and the maximum number of available observations at each time step $kT_{s}$ is 0 and 4, respectively. Again, the optimal window size of n=55 samples is considered. As expected and shown in Figure 9, reporting the usual RMSE of position and orientation, more observations generally leads to better accuracy.

What is instead of more relevance is the relatively high robustness of the filter to the lack of measurements: indeed even with 30–40% of the available measurements, the uncertainty growth remains acceptable and slightly worse than using all the measurements.

#### 4.1.4. Vehicle Dynamic

**Figure 10:**
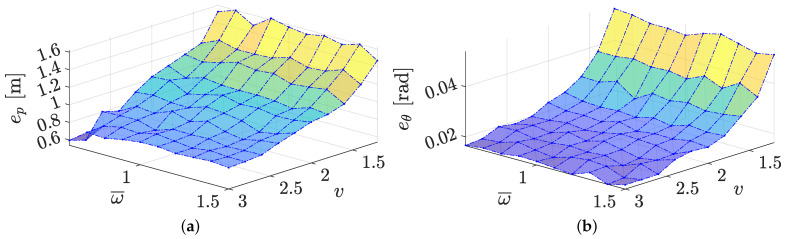
(**a**) Position and (**b**) orientation RMSE for different values of the angular and linear velocities.

In the last set of simulations, we investigate the impact of different linear and angular velocities on the localisation error using one single trajectory among the available set, which is represented in Figure 4 for the realistic warehouse scenario. Since different velocity profiles are used, ranging in the interval (2.8,1.5) m/s for the linear velocity and in the interval (|1.5|,|0.6|) rad/s for theangular velocity (we recall that the sign of the angular velocity is determined by the turn direction), different trajectories for the same sequence of via-points are generated (see Figure 4). For a more quantitative analysis, we consider sufficiently long trajectories, ranging from 70 to 164 s (the difference in the length are actually dictated by the different velocities on the same path). Results are reported in the RMSE plot of Figure 10, where we use the SEKF with the optimal window size of n=55 samples and we denote with $\bar{\omega }=\left|\omega \right|$, i.e., the magnitude of turning speed.

Both the position and the orientation RMSEs are reported, which are computed on Monte Carlo trials of 100 executions. Due to the presence of a Kalman filter estimator, it is expected that the RMSE reaches a steady state value determined by the model and measurement covariances. This is certainly the case for linear systems, while for nonlinear systems (as the one at hand) it is expected a fluctuation of the RMSE, induced by the particular trajectory followed. Nevertheless, Figure 10 shows a clear divergence when the forward velocity *v* decreases. This apparently strange behaviour is a direct consequence of the longer trajectory followed when the forward velocity reduces, which implies a higher number of integration steps of the model noises with the same variance in the filter prediction step. This is usually compensated by the larger amount of measurements that can be collected along the trajectory. Unfortunately, this is not the case for RFID-tag measurements, where the difference of ranges are actually collected (see Section 3.2). Hence, smaller forward velocities *v* imply smaller displacements between two time steps, thus smaller differences among the range measurements. Therefore, the observations become comparable with the measurement uncertainties, thus reducing the amount of information carried by each observation. This fact is further proved by the substantial independence of the RMSE by the angular velocity ω, which does not affect the ranging measurements by design (see Figure 10).

### 4.2. Experimental Results

The effectiveness of the proposed SEKF estimator is further substantiated by the experiments conducted on a robot moving in an indoor environment, as described in Section 3.3. The RFID reader has a nominal phase noise standard deviation of 0.1 rad due to thermal noise, meaning that the measured differences of distance ${\dot{d}}_{i,k}$ in (8) have a nominal standard deviation of around 4 mm. In practical situations, this modelled uncertainty is increased due to the presence of multi-path interference that leads to a higher value than the nominal one, but still acceptable. Despite the phase robustness once adopted for localisation, the problem of robot displacements between consecutive measurements has a detrimental effect (as noticed and analysed in simulations in Figure 10). In practice, if the ${\dot{d}}_{i,k}$ values are too close to zero, the relative impact of the measurement noises on the observation function is high. On the other hand, a relatively high sampling frequency is needed for reliable localisation using RFID signal phase to avoid problems due to the 2π-phase ambiguity. In other words, since the average spatial sampling (which depends on the robot speed and on the RFID system sampling rate) cannot be too large, a relative high number of tag reading for each time step must be guaranteed to compensate for the high relative impact of the measurement noise on the observation function. The variance of the uncertainties affecting vehicle velocities $v_{k}$ and $\omega_{k}$ has been obtained with a Type A analysis, according to the Guide to the Expression of Uncertainty in Measurement [45]. In particular, we have implemented an EKF with encoder readings and actual range measurements and compared against the ground truth range measurements, in order to derive the odometry covariance matrix. Next, we have tested the estimation procedure in simulation by running Monte Carlo experiments using a very large set of testing trajectories. At the end of the process, we found out that $\sigma_{v}=0.1$ m/s and $\sigma_{\omega }=0.05$ rad/s.

**Figure 11:**
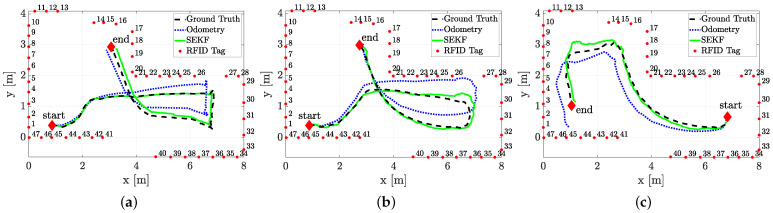
Three (**a**–**c**) sample trajectories of the robot (dashed line), SEKF estimates (solid line) and dead reckoning only reconstructed trajectories (dotted line). In all the pictures, the starting and ending positions of the trajectories as well as the RFID tag locations are reported as well.

Three different sample trajectories are considered in this section, and reported in Figure 11. The robot was driven manually through a remote control by an operator.

**Figure 12:**
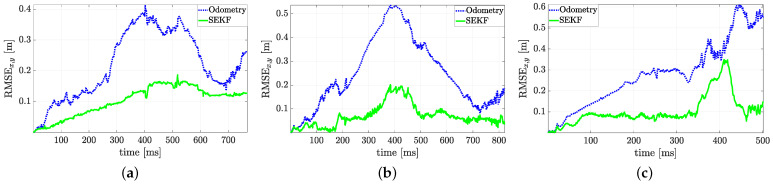
Position RMSE time evolution during localisation for (**a**) the sample trajectory of Figure 11a, (**b**) the sample trajectory of Figure 11b, (**c**) the sample trajectory of Figure 11c.

The trajectories estimated by the SEKF (solid lines) as well as the dead reckoning results reported for comparison (dotted lines, obtained using only the encoder measurements and the model (2)) and the ground truth data (dashed lines) are plotted in Figure 11. The estimated trajectories (both using the SEKF or the encoders only) assumes perfect knowledge of the initial conditions, which is a strong assumption for any Bayesian estimator. Nonetheless, the purpose of these experiments is just to test the effect of a reduced forward velocity, set to v=0.23 m/s, which has detrimental effect on the overall accuracy, as previously mentioned. From a qualitative analysis of Figure 11, it is evident how the SEKF returns a good path reconstruction, with a maximum error, happening in the turning conditions, which is approximately around 30 cm. A more detailed analysis of the localisation uncertainty for the three trajectories is reported in Figure 12, where the position RMSE shows to be almost everywhere less than 15 cm.

For the orientation RMSE we have along the trajectories approximately 0.2 rad.

**Figure 13:**
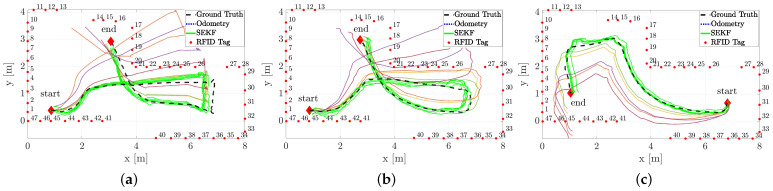
Trajectory estimation for (**a**) the sample trajectory of Figure 11a, (**b**) the sample trajectory of Figure 11b, (**c**) the sample trajectory of Figure 11c with different range of uncertainties about the true initial conditions.

To account for theinitial-condition uncertainty, Figure 13 reports the same trajectories shown in Figure 11 but assuming that the initial state ${\hat{s}}_{0}$ is randomly generated from a Gaussian distribution with zero-mean and covariance matrix $P_{0}^{\left(3,3\right)}$ (i.e., the marginal of the filter covariance matrix $P_{0}$ in (15) restricted to the robot state ${\hat{s}}_{k}$).

**Figure 14:**
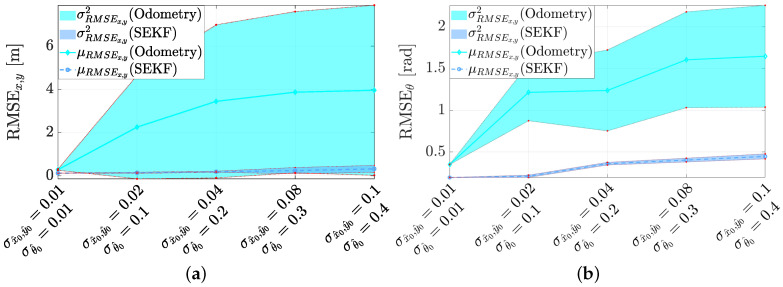
Localisation estimation accuracy in presence of different uncertainties about the true initial conditions for (**a**) the position and (**b**) the orientation.

Moreover, the roots of the elements on the diagonal of $P_{0}^{\left(3,3\right)}$, i.e., the standard deviations, have been changed as reported in Table 1. As can be seen, the initial conditions slightly modify the reconstructed trajectory, thus showing the robustness and the consistency of the SEKF. The impact of the initial condition uncertainty is also analysed in a quantitative manner in Figure 14.

The results have been collected by executing a Monte Carlo approach for the different initial conditions and using the same experimental data. In particular, we performed 15,000 executions for each of the initial $P_{0}^{\left(3,3\right)}$ reported in Table 1. It can be fairly observed from the corresponding graphs that, the localisation accuracy is insensitive from the initial-condition uncertainty, being the slight increase of the RMSE dictated by the first part of the trajectories (i.e., a transient effect). To assess the validity of the proposed RMSE analysis, we show also the odometry-only reconstruction that obviously report a direct proportionality with the initial conditions (i.e., the uncertainty in the initial pose is not transient).

**Figure 15:**
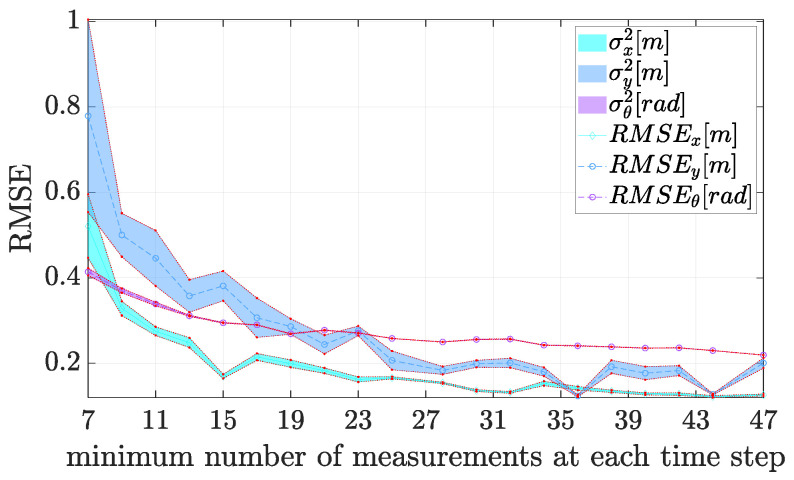
Impact of minimum number of available phase measurements at each time step on the localisation accuracy for the current experimental setup.

As a final empirical analysis on the experimental set-up, we analyse the problem of the number of RFID tag measurements. Intuitively, the larger is the number of sensed tags at each time step, the lower will be the RMSE. Indeed, this is empirically verified in Figure 15 considering the RMSE for the *x*, *y* and θ quantities and the associated standard deviations.

These results have be collected considering the average robot speed of 0.23 m/s and considering the odometry sampling period $T_{s}=100$ ms as the main sampling time (during the experiments, we used fixed time steps and the assumption of constant velocities at each time step). As can be seen from the figure, relying on a few number of tags, has resulted in a high localisation uncertainty, as expected. From the picture, just 10 tags provide a sufficiently accurate SEKF, at least for the purposes of trajectory analysis in the industrial domain. In an actual deployment, hence, it could be beneficial to increase the number of RFID antennas and the sampling frequency as well. Nevertheless, in such scenarios, other second-order effects will take place at the cost of a degradation in the accuracy of phase measurements, therefore such a solution is discouraged with the actual equipment. To summarise, we can conclude that it is highly recommended to have access to an average number of 7 to 10 tags readings at each time step. Of course, this is an average rate, since in the actual applications absence of measurements at some time steps cannot be ruled out, but, as reported in Figure 9, this phenomenon slightly affects the SEKF.

## 5. Discussion

The focus of the presented solution is to determine the best possible estimate of the whole trajectory followed by the agent inside a plant using an EKF-based smoother rather than an EKF. This issue turns to be very relevant in an actual industrial application where the knowledge of set of trajectories followed by the different moving agents is extremely important to detect points of failures as well as to optimise the team trajectories as a whole. As discussed in the Introduction, different RFID-based localisation systems were designed in the literature. Although some solutions rely on the combination of phase and amplitude measurements [26,33], phase-only measurements prove to be quite effective in indoor environments [21]. To account for the phase ambiguity, different filters were provided that are based on the fusion with wheel encoders, ranging from Unscented Kalman Filters (UKF) [37], Multi-Hypothesis EKF [32] and particle filters [35].

Nevertheless, this paper marks a non negligible difference since it proposes a smoother EKF-RTS to deal with the peculiarity of the phase measurements, which inherently need measurement differences to retrieve useful quantities. In particular, we presented an EKF-RTS smoother to reconstruct the whole trajectory of a unicycle-like vehicle moving in an industrial environment. The analysis of the uncertainty affecting the estimates in a realistic warehouse scenario with only four RFID tags is presented and compared with a standard EKF approach. We have also proved that the smoother may be endowed with a tuning parameter, i.e., the length of the observation window *n*, whose value leverages between the performance of the filter and the computational requirements. Another important feature that is totally missed in the cited literature, is related to the robustness of the solution when measurements are missing, which again marks a notable performance improvement with respect to a filter-based solution, i.e., the EKF. We have additionally explicitly accounted for the relative robot displacement between consecutive RFID tag phase readings, which may jeopardise the solution performance. This tradeoff was also analysed and exposed in the experimental section, where on-field results have been collected and a specific collection of data has been conceived and reported.

To summarise, the proposed smoother-based solution proved to be more effective of a standard filter and demonstrated its viability, effectiveness and feasibility in a real-time scenario by means of the tuning parameter *n*. The shortcomings and the advantages of the proposed solution have been throughly exposed and discussed with simulations and experiments.

## Figures and Tables

**Table 1:** Standard deviations assumed for the initial robot state and distributed according to a Gaussian probability density function.

	${\hat{x}}_{0}$	${\hat{y}}_{0}$	${\hat{\theta }}_{0}$
σ	0.01	0.01	0.01
	0.02	0.02	0.1
	0.04	0.04	0.2
	0.08	0.08	0.3
	0.1	0.1	0.4

## Abbreviations

The following abbreviations are used in this manuscript:

EKF	Extended Kalman Filter
EKF-RTS	Extended Kalman Filter Rauch-Tung-Striebel smoother
LiDAR	Laser Imaging Detection and Ranging
RF	Radio Frequency
RFID	Radio Frequency IDentification
SEKF	Fixed-lag Extended Kalman Filter Rauch-Tung-Striebel smoother
SEKF${}^{2}$	Full-length Extended Kalman Filter Rauch-Tung-Striebel smoother
UHF	Ultra-High-Frequency
UKF	Unscented Kalman Filter
UWB	Ultra Wide Band

## References

[1] Drath R., Horch A. (2014). Industrie 4.0: Hit or Hype? [Industry Forum]. IEEE Ind. Electron. Mag..

[2] Batalla J.M., Mavromoustakis C.X., Mastorakis G., Xiong N.N., Wozniak J. (2020). Adaptive Positioning Systems Based on Multiple Wireless Interfaces for Industrial IoT in Harsh Manufacturing Environments. IEEE J. Sel. Areas Commun..

[3] Kulyukin V., Kutiyanawala A., LoPresti E., Matthews J., Simpson R. iWalker: Toward a Rollator-Mounted Wayfinding System for the Elderly. Proceedings of the IEEE International Conference on RFID.

[4] Nazemzadeh P., Moro F., Fontanelli D., Macii D., Palopoli L. (2015). Indoor Positioning of a Robotic Walking Assistant for Large Public Environments. IEEE Trans. Instrum. Meas..

[5] Gallant M.J., Marshall J.A. (2016). Two-Dimensional Axis Mapping Using LiDAR. IEEE Trans. Robot..

[6] Mautz R., Tilch S. Survey of optical indoor positioning systems. Proceedings of the International Conference on Indoor Positioning and Indoor Navigation (IPIN).

[7] Nazemzadeh P., Fontanelli D., Macii D., Palopoli L. (2017). Indoor Localization of Mobile Robots through QR Code Detection and Dead Reckoning Data Fusion. IEEE/ASME Trans. Mechatron..

[8] Ijaz F., Yang H.K., Ahmad A., Lee C. Indoor positioning: A review of indoor ultrasonic positioning systems. Proceedings of the 15th International Conference on Advanced Communication Technology (ICACT).

[9] Li C., Mo L., Zhang D. (2019). Review on UHF RFID Localization methods. IEEE J. Radio Freq. Identif..

[10] Chen P., Xu Y.B., Chen L., Deng Z.A. (2013). Survey of WLAN Fingerprinting Positioning System. Appl. Mech. Mater..

[11] Giovanelli D., Farella E., Fontanelli D., Macii D. Bluetooth-based Indoor Positioning through ToF and RSSI Data Fusion. Proceedings of the International Conference on Indoor Positioning and Indoor Navigation (IPIN).

[12] Jiménez Ruiz A.R., Seco Granja F. (2017). Comparing Ubisense, BeSpoon, and DecaWave UWB Location Systems: Indoor Performance Analysis. IEEE Trans. Instrum. Meas..

[13] Magnago V., Corbalán P., Picco G., Palopoli L., Fontanelli D. Robot Localization via Odometry-assisted Ultra-wideband Ranging with Stochastic Guarantees. Proceedings of the IEEE/RSJ International Conference on Intelligent Robots and System (IROS).

[14] Motroni A., Nepa P., Magnago V., Buffi A., Tellini B., Fontanelli D., Macii D. SAR-based Indoor Localization of UHF-RFID Tags via Mobile Robot. Proceedings of the International Conference on Indoor Positioning and Indoor Navigation (IPIN).

[15] Buffi A., Fontanelli D., Macii D., Magnago V., Motroni A., Nepa P., Tellini B. UHF-RFID Localization: The Problem of Antenna Phase Center in Phase-based Methods. Proceedings of the 2019 13th European Conference on Antennas and Propagation (EuCAP).

[16] Sarkka S., Viikari V.V., Huusko M., Jaakkola K. (2012). Phase-Based UHF RFID Tracking With Nonlinear Kalman Filtering and Smoothing. IEEE Sens. J..

[17] Yang L., Cao J., Zhu W., Tang S. (2015). Accurate and Efficient Object Tracking Based on Passive RFID. IEEE Trans. Mob. Comput..

[18] Martinelli F. (2019). Simultaneous Localization and Mapping Using the Phase of Passive UHF-RFID Signals. J. Intell. Robot. Syst..

[19] Park S., Lee H. (2013). Self-Recognition of Vehicle Position Using UHF Passive RFID Tags. IEEE Trans. Ind. Electron..

[20] Motroni A., Buffi A., Nepa P., Tellini B. (2020). Sensor-Fusion and Tracking Method for Indoor Vehicles with Low-Density UHF-RFID Tags. IEEE Trans. Instrum. Meas..

[21] Bernardini F., Buffi A., Motroni A., Nepa P., Tellini B., Tripicchio P., Unetti M. (2020). Particle Swarm Optimization in SAR-based Method enabling Real-Time 3D Positioning of UHF-RFID Tags. IEEE J. Radio Freq. Identif..

[22] Motroni A., Buffi A., Nepa P. (2020). A survey on Indoor Vehicle Localization through RFID Technology. IEEE Access.

[23] Duan C., Rao X., Yang L., Liu Y. Fusing RFID and computer vision for fine-grained object tracking. Proceedings of the IEEE INFOCOM 2017—IEEE Conference on Computer Communications.

[24] Shamsfakhr F., Palopoli L., Fontanelli D., Motroni A., Buffi A. Robot Localisation using UHF-RFID Tags for Industrial IoT Applications. Proceedings of the 2020 IEEE International Workshop on Metrology for Industry 4.0 IoT.

[25] Motroni A., Nepa P., Buffi A., Tellini B. A Phase-Based Method for Mobile Node Localization through UHF-RFID Passive Tags. Proceedings of the 2019 IEEE International Conference on RFID Technology and Applications (RFID-TA).

[26] Pomárico-Franquiz J.J., Shmaliy Y.S. (2014). Accurate Self-Localization in RFID Tag Information Grids Using FIR Filtering. IEEE Trans. Ind. Inform..

[27] Gueaieb W., Miah M.S. Mobile robot navigation using particle swarm optimization and noisy RFID communication. Proceedings of the 2008 IEEE International Conference on Computational Intelligence for Measurement Systems and Applications.

[28] Nikitin P.V., Martinez R., Ramamurthy S., Leland H., Spiess G., Rao K.V.S. Phase based spatial identification of UHF RFID tags. Proceedings of the 2010 IEEE International Conference on RFID (IEEE RFID 2010).

[29] Buffi A., Nepa P., Lombardini F. (2015). A Phase-Based Technique for Localization of UHF-RFID Tags Moving on a Conveyor Belt: Performance Analysis and Test-Case Measurements. IEEE Sens. J..

[30] Tzitzis A., Megalou S., Siachalou S., Tsardoulias E., Filotheou A., Yioultsis T., Dimitriou A.G. (2020). Trajectory Planning of a Moving Robot Empowers 3D Localization of RFID Tags with a Single Antenna. IEEE J. Radio Freq. Identif..

[31] Buffi A., Motroni A., Nepa P., Tellini B., Cioni R. (2019). A SAR-Based Measurement Method for Passive-Tag Positioning With a Flying UHF-RFID Reader. IEEE Trans. Instrum. Meas..

[32] DiGiampaolo E., Martinelli F. (2014). Mobile Robot Localization Using the Phase of Passive UHF RFID Signals. IEEE Trans. Ind. Electron..

[33] Martinelli F. (2015). A Robot Localization System Combining RSSI and Phase Shift in UHF-RFID Signals. IEEE Trans. Control Syst. Technol..

[34] Buffi A., Nepa P., Motroni A., Tellini B. (2020). Mobile Device Self-Location Method Using at Least One Passive Radio-Frequency Device.

[35] Tao B., Wu H., Gong Z., Yin Z., Ding H. (2020). An RFID-Based Mobile Robot Localization Method Combining Phase Difference and Readability. IEEE Trans. Autom. Sci. Eng..

[36] Magnago V., Palopoli L., Fontanelli D., Macii D., Motroni A., Nepa P., Buffi A., Tellini B. Robot Localisation based on Phase Measures of backscattered UHF-RFID Signals. Proceedings of the 2019 IEEE International Instrumentation and Measurement Technology Conference (I2MTC).

[37] Magnago V., Palopoli L., Buffi A., Tellini B., Motroni A., Nepa P., Macii D., Fontanelli D. (2020). Ranging-free UHF-RFID Robot Positioning through Phase Measurements of Passive Tags. IEEE Trans. Instrum. Meas..

[38] Nepa P., Motroni A., Congi A., Ferro E.M., Pesi M., Giorgi G., Buffi A., Lazzarotti M., Bellucci J., Galigani S. I-READ 4.0: Internet-of-READers for an efficient asset management in large warehouses with high stock rotation index. Proceedings of the 2019 IEEE 5th International Forum on Research and Technology for Society and Industry (RTSI).

[39] Magnago V., Palopoli L., Passerone R., Fontanelli D., Macii D. (2019). Effective Landmark Placement for Robot Indoor Localization with Position Uncertainty Constraints. IEEE Trans. Instrum. Meas..

[40] Palopoli L., Macii D., Fontanelli D. A Positioning Filter based on Uncertainty and Observability Analyses for Nonholonomic Robots. Proceedings of the 2020 IEEE International Instrumentation and Measurement Technology Conference (I2MTC).

[41] Simon D. (2006). Optimal State Estimation: Kalman, H Infinity, and Nonlinear Approaches.

[42] Nassar S., Niu X., El-Sheimy N. (2007). Land-vehicle INS/GPS accurate positioning during GPS signal blockage periods. J. Surv. Eng..

[43] Magnago V., Andreetto M., Divan S., Fontanelli D., Palopoli L. Ruling the Control Authority of a Service Robot based on Information Precision. Proceedings of the IEEE International Conference on Robotics and Automation (ICRA).

[44] Andreetto M., Divan S., Ferrari F., Fontanelli D., Palopoli L., Zenatti F. (2018). Simulating passivity for Robotic Walkers via Authority-Sharing. IEEE Robot. Autom. Lett..

[45] (1993). Guide to the Expression of Uncertainty in Measurement.

